# Light quality and time in shelter modulate behavior and cortisol in the domestic cat (*Felis catus*)

**DOI:** 10.1016/j.isci.2025.112709

**Published:** 2025-05-20

**Authors:** Alexandra M. Yaw, Mary E. Gardella, Jacquelyn Jacobs, Hanne M. Hoffmann

**Affiliations:** 1 Department of Animal Science, Michigan State University, East Lansing, MI 48824, USA

**Keywords:** Molecular biology, Neuroscience

## Abstract

Light is a principal synchronizer of behavior, circadian rhythms, and hormone release patterns, including stress responses. High stress in domestic cats (*Felis catus*) increases risk of disease, promotes undesirable behaviors, and is associated with reduced adoptions from shelters. To determine how light properties impact stress and behavioral activity patterns in cats, the effects of light intensity and composition were tested in 101 male and female cats using standard, dim, and dim, blue-depleted light during the initial five days in a shelter environment. Cats exhibited circadian locomotor activity, peaking at lights on, in all light conditions. Cortisol levels decreased under dim, blue-depleted light versus standard light on day 5. Probability of hiding was only increased in the dim light condition. A behavioral approach test showed declined behavioral stress markers over time in the shelter. Here, we demonstrate that indoor light conditions and length of time in shelter modulate stress in cats.

## Highlights

• Light intensity, light composition, and time in shelter modulated urinary cortisol levels
• Behavioral markers of stress in cats decreased over the first five days in the shelter
• All cats had circadian activity patterns, with peak activity when the lights turned on

## Graphical abstract

**Figure:**
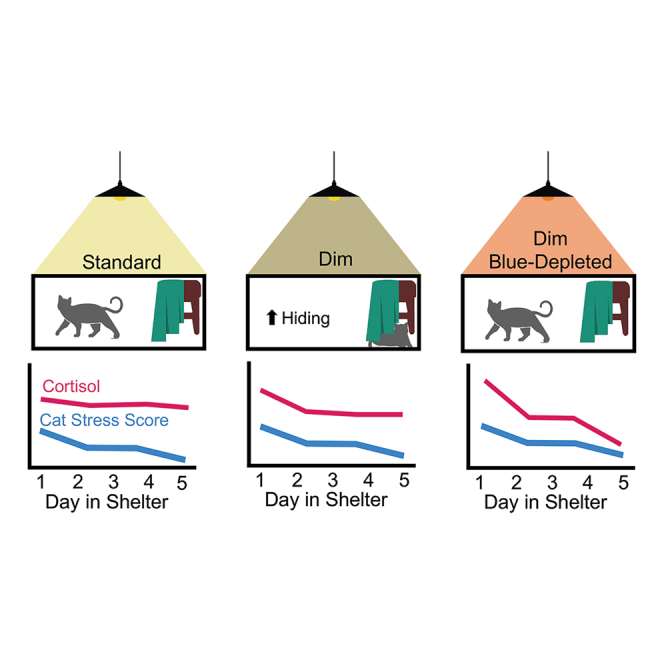

## Introduction

As organisms evolved to the Earth’s 24-h day/night cycles, species have adapted to fit temporal niches.^1^ The timing of behaviors and physiological functions to specific times of day not only aids survival by predicting changes in the environment, reducing predation, and increasing reproductive success, but also contributes to overall health. Light is a primary signal that entrains circadian rhythms in mammals and is a complex variable comprising several qualities, including intensity, spectral composition (wavelength), duration (photoperiod), and time of day of exposure. Each quality contributes to an organism’s ability to synchronize physiological functions and behaviors to external time of day.^2^^,^^3^^,^^4^^,^^5^^,^^6^ Not only are light qualities essential to the maintenance of robust circadian rhythms, but in mammals, both light intensity and composition are established modulators of mood, stress responses, sleep-wake cycles, and locomotor activity.^2^^,^^7^^,^^8^^,^^9^^,^^10^^,^^11^^,^^12^^,^^13^ Many mammals, including humans and laboratory rodents, respond to light properties such that bright light and short (blue) wavelengths act as more powerful stimulatory cues to entrain circadian rhythms compared to dim light and longer (red) wavelengths.^14^ While light has well-established roles modulating circadian rhythms, including stress responses, the impact of light on animal wellbeing in captive environments has been understudied.^15^^,^^16^

The domestic cat (*Felis catus*) is a species of particular interest due to their roles as companion animals and biomedical research subjects for translational and veterinary studies.^17^^,^^18^ Additionally, domestic cats have a high degree of conserved genetic and behavioral similarities with wild cats in the Felidae family.^19^^,^^20^^,^^21^ Domestic cats are used as a research model for wild cats, commonly focused on reproduction, which has led to essential insights for overcoming breeding difficulties in wild felid species.^21^^,^^22^^,^^23^ Not only is understating how domestic cats respond to light important for informing housing conditions in research facilities and shelters, but this information can also provide a starting point for determining the impacts of light qualities on other Felidae species housed in captivity and may be of particular relevance for Felidae species that are involved to conservation breeding programs.

Stress is broadly defined as actual or anticipated threats to wellbeing and/or threats to the disruption of homeostasis.^24^ In domestic cats, high stress is linked to increased health risks, increased aggressive and compulsive behaviors^25^ and reduced adoptions.^26^ Stress-related information is relayed to the brain via sensory input, which in turn relays neural and endocrine signals to adapt to a perceived threat or homeostatic imbalance.^24^ These adaptations include both autonomic nervous system responses, which provide immediate reflexive responses to stressor exposures, and hypothalamic-pituitary-adrenocortical axis activation, which results in neuroendocrine release of glucocorticoids. Light information modulates autonomic nervous system responses in cats, including autonomic nervous system–driven pupillary reflexes;^27^^,^^28^ however, our understating of light’s impact on hypothalamic-pituitary-adrenocortical activation in domestic cats is less established. In other mammals, such as humans and laboratory rodents, light information is translated from the eye to the suprachiasmatic nucleus,^14^^,^^29^ a brain region essential for coordinating timekeeping and circadian rhythms. The suprachiasmatic nucleus then projects to the paraventricular nucleus, which is a major stress regulation center,^30^ providing a link between light and hypothalamic-pituitary-adrenocortical axis stress responses. In domestic cats, light is translated from the eye to the suprachiasmatic nucleus,^29^ but the connections between light, stress, and circadian rhythms in this species are still not well understood.

In terms of circadian timekeeping, the temporal niche of the domestic cat is highly variable, with reports of domestic cats exhibiting circadian^1^^,^^31^ and polyphasic^32^^,^^33^ and a lack of circadian rhythms.^32^ When domestic cats display circadian rhythms, multiple chronotypes (diurnal, nocturnal, and crepuscular)^31^^,^^32^^,^^34^^,^^35^^,^^36^^,^^37^^,^^38^ are found, suggesting that the environment strongly influences the expression of locomotor activity patterns in cats.^39^ It is currently hypothesized that cats exhibit weak endogenous circadian activity rhythms that are highly entrainable to environmental conditions, including social interactions with humans and conspecifics, food availability, and light.^34^^,^^35^^,^^40^ Despite this knowledge, the capacity of light qualities to modulate physiology and behavior in domestic cats remains largely unknown. The Association of Shelter Veterinarians’ Guidelines for Standards of Care in Animal Shelters recommends lighting that promotes a safe work environment for the staff and effective observation of animals.^41^ This recommendation suggests designing facilities to incorporate as much natural sunlight as possible and, if artificial lighting is necessary, to approximate natural light duration and intensity. These guidelines are largely based on research in non-feline mammals, primarily humans and rodents, that have well characterized circadian (24h) rhythms that are strongly modulated by light.^41^^,^^42^^,^^43^ However, it is important to note that indoor artificial lighting is very different from sunlight, where indoor lighting intensity is much lower and has a reduced spectrum of emitted wavelengths.^44^^,^^45^ Increasing our understanding of how light properties modulate domestic cat circadian rhythms and stress in captive environments, such as animal rescues, shelters, catteries, and research laboratories, is important for both animal welfare and academic rigor in veterinary and biomedical research.

Given the importance of light in regulating circadian rhythms and stress, our goals were to (1) determine how light intensity and composition modulate cat stress and (2) characterize behavioral circadian rhythms of cats in a shelter environment in response to the studied light conditions. We hypothesized that reducing room light intensity and removing blue light wavelengths would reduce cat stress compared to standard white, LED lighting.

## Results

### Light conditions and time in the shelter impact cortisol, hiding, and overall activity levels

**Figure 1:**
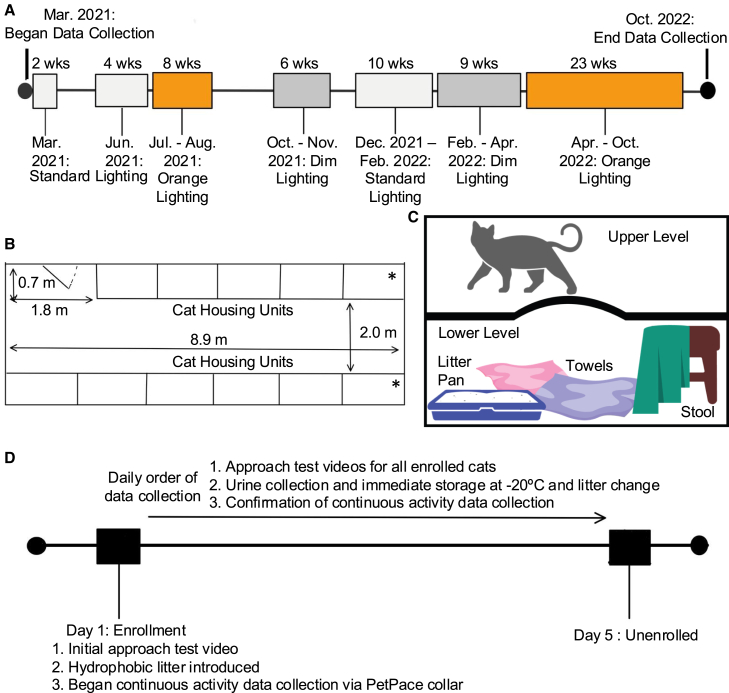
Diagrams of experimental timelines and shelter housing (A) Experimental timeline of data collection and lighting conditions over 19 months. Box color indicates lighting conditions with light gray for standard lighting, dark gray for dim lighting, and orange for dim, blue-depleted lighting. (B) The dimensions and layout for the shelter housing room and cat housing units. Smaller cat housing units are indicated by “∗” and the location of the door to enter the room is indicated on the upper left. (C) Individual cat units comprised two levels that cats were able to freely move between. The upper level was left empty and a litter pan, towels, and a stool covered with a towel (covered hiding spot) were placed on the lower level. (D) Cats were enrolled into the study between 1300 and 1700h within the first 24h after they arrived at the shelter. Following enrollment, daily data for the approach test, urine collection, and PetPace data monitoring were collected through the date of unenrollment.

While the impacts of light quality on domestic cats are largely unknown, light quality has begun to emerge as a new target for improving the welfare of animals used for production and in zoo and aquarium settings.^15^^,^^46^^,^^47^^,^^48^ In collaboration with a Midwestern animal control and shelter, we evaluated the impact of three light conditions on intact cats admitted to the shelter over 19 months (Figure 1A). As multiple aspects of light quality are known to modulate physiology and behavior,^3^^,^^4^^,^^5^^,^^6^^,^^14^^,^^49^ we analyzed both light intensity and composition in three types of lighting (standard, dim, and dim, blue-depleted; the latter will be referred to as orange based on the human perception of the blue-depleted light in the cat holding room (Figures 1B and 1C).

**Figure 2:**
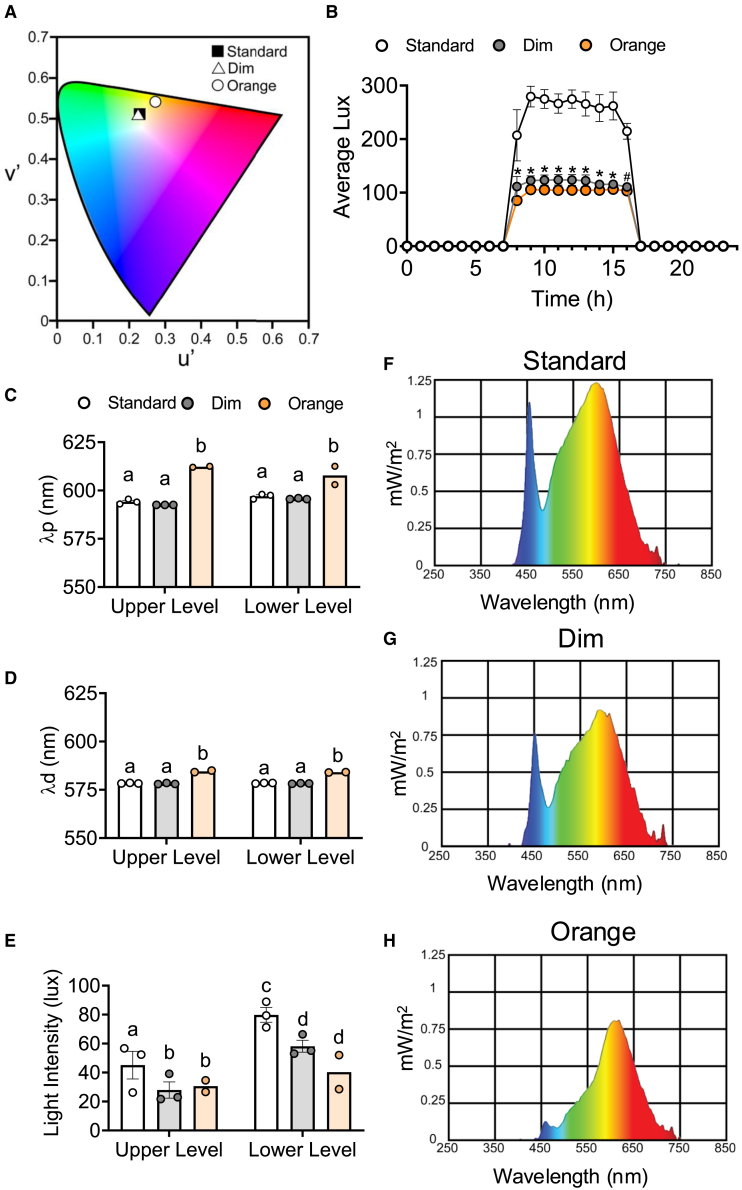
Cat unit lighting conditions for standard, dim and orange overhead lighting (A) CIELUV 1976 chromaticity diagram displaying average cat unit perceived hue. Standard light is indicated by black square, dim light is indicated by white triangle, and orange light is indicated by white circle. (B) Average cat room light intensity (lux) in standard, dim, and orange light conditions across time of day. “∗” indicates significant differences (*p* < 0.05) between both dim and orange light compared to standard and “#” indicates significant differences (*p* < 0.05) between orange and standard light. (C) Average peak wavelength (λp), (D) dominant wavelength (λd), and (E) light intensity from inside cat units were analyzed via two-way Analysis of Variance (n = 2–3 per light condition). Independent light measures are shown as circles, with white indicating standard light, gray indicating dim light, and orange indicating dim, blue-depleted (orange perceived) light. Data are represented as mean ± SEM. Different letters indicate significant differences, *p* < 0.05. Spectral power distribution for (F) standard, (G) dim, and (H) orange conditions.

To determine how standard, dim, and orange lighting altered physiological stress levels, we collected daily urinary cortisol for at least three days in 43 cats (Figure 1D). Unaltered shelter overhead LED lighting (standard) was used as the control. Both dim and orange (dim, blue-depleted) lighting conditions were achieved through attaching light filters to the overhead lighting. Blue wavelengths (orange condition) were targeted for their importance in regulating both circadian rhythms and mood in mammals.^50^^,^^51^^,^^52^ Differences in the type of light are reflected in the representative spectral power distributions, 24h room light intensity, in-unit peak and dominant wavelengths, and in-unit intensity (Figures 2A–2H). As the light filters for the orange condition significantly reduced the light intensity of the room (Figures 2B and 2E), and light intensity also impacts circadian rhythms and stress,^4^^,^^5^^,^^53^^,^^54^^,^^55^ a dim light condition was included. There was a significant interaction of light condition and time in day (hours) on room light intensity, F(46, 138) = 22.33; *p* < 0.0001 (Figure 2B). Room light intensity did not differ during times of lights off (0000–0700h and 1700–2300h), but both dim and orange light had reduced intensity compared to standard light from 0800h to 1500h, while only orange differed from standard light at 1600h. Inside the cat units, orange lighting had a reduced peak in the blue-light portion (450–495nm; Figures 2F–2H) that is not present in standard or dim spectral power distributions, and standard light intensity (1.24 mW/m^2^) is higher than both dim (0.93 mW/m^2^) and orange light (0.82 mW/m^2^).

**Figure 3:**
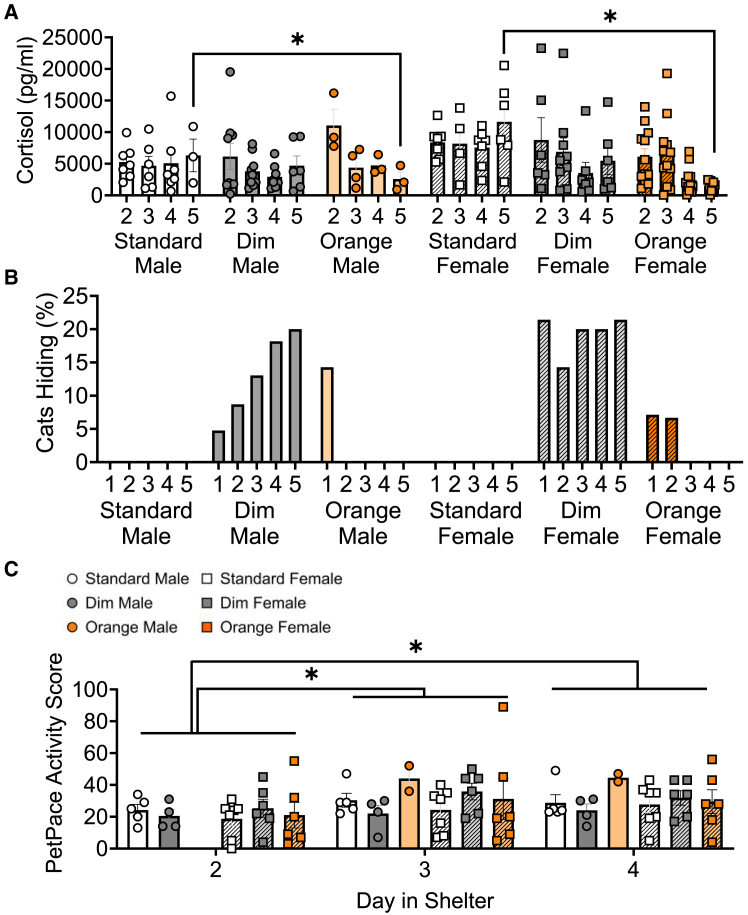
Light and/or time in shelter impact cortisol, hiding behavior, and activity levels in shelter cats (A) Male and female cat urinary cortisol by day in shelter in standard, dim, and orange overhead lighting were analyzed via mixed model ANOVA (Type 3 tests, KR-method) for sex, day in shelter and sex differences, n = 4–12 per group. Data are represented as mean ± SEM. (B) Percentage of male and female cats hiding during daily care by day in shelter in standard, dim, and orange lighting. Probability of hiding was analyzed via Bayesian logistic regression model, n = 4–19 per group. (C) Male and female PetPace Activity scores by day in shelter in standard, dim, and orange lighting were analyzed via mixed model ANOVA (Type 3 tests, KR-method) for sex, day in shelter and sex differences, n = 2–7 per group. Individual cats are represented by circles with color indicating light condition. Data are represented as mean ± SEM. “∗” indicates significant differences with *p* < 0.05.

As previous studies have established that stress levels decrease across days as cats habituate to a new environment^56^^,^^57^^,^^58^ and that sex differences are found in many species in both stress responses^59^ and activity patterns,^60^^,^^61^^,^^62^ we included day in the shelter and sex as variables in addition to light condition. There were no significant three-way interactions for urinary cortisol or PetPace activity scores (mixed model ANOVA, Table 1). Urinary cortisol levels (Figure 3A) were dependent on both light condition and day in shelter (F[6,100.43] = 2.22, *n* = 15–20 per light condition, *p* = 0.047). Cats in orange light on day 5 (1730 ± 349 pg/mL) had reduced cortisol (*p* = 0.019) compared to cats in standard lighting (9861 ± 2093 pg/mL). In addition to cortisol levels, we measured cat hiding behavior (Figure 3B), an established behavioral response to stress in cats.^63^^,^^64^ Bayesian logistic regression with random intercepts for each individual cat to account for repeated measures (Table 2) was used to predict the probability of hiding behavior as a function of sex, light condition, and day (Table 3). Under standard and orange light, the predicted probabilities of hiding were low (standard: 0.00–0.002; orange: <0.002), with most estimates close to zero. Hiding probabilities were higher under dim lighting, with male cats demonstrating increased probabilities across days, peaking at day 4 (Estimate = 0.013, 95% Cl: [0, 0.083]) and day 5 (Estimate = 0.011, 95% Cl: [0, 0.087]). Female cats also had higher probabilities of hiding under dim light, in particular on day 5 (Estimate = 0.029, 95% Cl: [0, 0.191]), suggesting increased hiding in dim light, especially during later days in the shelter.

**Table 1:** Mixed model ANOVA table (Type 3 tests, KR-method) for sex, day in shelter, and sex differences for cortisol (n = 4–12 per group), and PetPace activity score (n = 2–7 per group) for each light condition

Variable	Effect	df	F	*p* value
Cortisol	Sex:Light:Day	6, 100.43	1.12	0.354
	**Light:Day**	**6, 100.43**	**2.22**	**0.047**
	Sex:Day	3, 100.53	1.04	0.378
	Sex:Light	2, 44.57	1.87	0.166
	**Day**	**3, 100.53**	**6.30**	**<0.001**
	Light	2, 44.57	1.42	0.252
	Sex	1, 44.51	2.27	0.139
PetPace Activity Score	Sex:Light:Day	3, 47	0.54	0.655
	Light:Day	4, 47	0.08	0.989
	Sex:Day	2, 47	0.48	0.620
	Sex:Light	2, 25.16	1.47	0.249
	**Day**	**2, 47**	**5.68**	**0.006**
	Light	2, 24.8	0.87	0.431
	Sex	1, 25.05	0.28	0.604

Significant data (p < 0.05.) are bolded.

**Table 2:** Bayesian logistic regression model of posterior mean estimate, standard error, 95% credible interval (CI) and R-hat statisic for all main effects and interactions between sex, light condition, and day in shelter, as well as random intercepts for each individual cat to account for repeated measures in cat hiding behavior (n = 4–19 per group)

Term	Estimate	Est. Error	95% CI (Lower)	95% CI (Upper)	R-hat
Intercept	−10.06	2.10	−14.57	−6.49	1.00
Sex Female (SexF)	0.28	1.47	−2.65	3.10	1.00
**Light Dim**	**2.51**	**1.46**	**−0.34**	**5.34**	**1.00**
Light Orange	0.46	1.59	−2.68	3.48	1.00
Day 2	−0.62	1.31	−3.21	1.87	1.00
Day 3	−0.51	1.36	−3.15	2.12	1.00
Day 4	−0.08	1.36	−2.74	2.59	1.00
Day 5	−0.09	1.40	−2.85	2.52	1.00
SexF:Light Dim	1.68	1.57	−1.49	4.79	1.00
SexF:Light Orange	0.15	1.69	−3.17	3.45	1.00
SexF:Day 2	0.19	1.44	−2.69	2.98	1.00
SexF:Day 3	−0.73	1.49	−3.81	2.17	1.00
SexF:Day 4	−1.20	1.49	−4.19	1.73	1.00
SexF:Day 5	−0.88	1.53	−3.91	2.02	1.00
Light Dim:Day 2	0.16	1.38	−2.52	2.89	1.00
Light Orange:Day 2	−0.14	1.58	−3.21	2.98	1.00
Light Dim:Day 3	1.36	1.39	−1.34	4.16	1.00
Light Orange:Day 3	−1.26	1.73	−4.74	2.06	1.00
Light Dim:Day 4	1.96	1.41	−0.79	4.83	1.00
Light Orange:Day 4	−1.37	1.68	−4.74	1.86	1.00
Light Dim:Day 5	1.52	1.48	−1.30	4.43	1.00
Light Orange:Day 5	−1.06	1.69	−4.45	2.25	1.00
SexF:Light Dim:Day 2	−0.32	1.56	−3.26	2.83	1.00
SexF:Light Orange:Day 2	0.91	1.67	−2.45	4.24	1.00
SexF:Light Dim:Day 3	0.16	1.55	−2.87	3.24	1.00
SexF:Light Orange:Day 3	−0.73	1.84	−4.46	2.88	1.00
SexF:Light Dim:Day 4	−0.31	1.52	−3.28	2.68	1.00
SexF:Light Orange:Day 4	−0.67	1.84	−4.34	2.84	1.00
SexF:Light Dim:Day 5	0.15	1.58	−2.92	3.33	1.00
SexF:Light Orange:Day 5	−0.81	1.85	−4.43	2.81	1.00

The reference group for the intercept was male cats on day 1 in standard light.

**Table 3:** Predicted probabilities and mean estimates, standard errors, and 95% credible intervals (Cl) of hiding for each combination of sex, light condition, and day in shelter using the Bayesian logistic regression model’s posterior distributions (n = 4–19 per group)

Sex	Light	Day	Estimate	Est. Error	2.5% CI	97.5% CI
M	Standard	Day 1	0.000	0.00058	0.000	0.002
M	Standard	Day 2	0.000	0.00076	0.000	0.002
M	Standard	Day 3	0.000	0.00072	0.000	0.001
M	Standard	Day 4	0.000	0.00098	0.000	0.002
M	Standard	Day 5	0.000	0.00116	0.000	0.003
F	Standard	Day 1	0.000	0.00096	0.000	0.002
F	Standard	Day 2	0.000	0.00251	0.000	0.004
F	Standard	Day 3	0.000	0.00093	0.000	0.002
F	Standard	Day 4	0.000	0.00116	0.000	0.002
F	Standard	Day 5	0.000	0.00283	0.000	0.002
M	Dim	Day 1	0.002	0.00513	0.000	0.014
M	Dim	Day 2	0.002	0.00557	0.000	0.014
M	Dim	Day 3	0.006	0.01255	0.000	0.037
M	Dim	Day 4	0.013	0.02650	0.000	0.083
M	Dim	Day 5	0.011	0.02737	0.000	0.087
F	Dim	Day 1	0.014	0.02898	0.000	0.092
F	Dim	Day 2	0.009	0.02136	0.000	0.059
F	Dim	Day 3	0.018	0.03478	0.000	0.118
F	Dim	Day 4	0.020	0.03790	0.000	0.131
F	Dim	Day 5	0.029	0.05622	0.000	0.191
M	Orange	Day 1	0.001	0.00185	0.000	0.004
M	Orange	Day 2	0.001	0.00334	0.000	0.004
M	Orange	Day 3	0.000	0.00136	0.000	0.002
M	Orange	Day 4	0.000	0.00206	0.000	0.002
M	Orange	Day 5	0.000	0.00280	0.000	0.003
F	Orange	Day 1	0.001	0.00439	0.000	0.010
F	Orange	Day 2	0.002	0.00838	0.000	0.017
F	Orange	Day 3	0.000	0.00138	0.000	0.002
F	Orange	Day 4	0.000	0.00120	0.000	0.002
F	Orange	Day 5	0.000	0.00207	0.000	0.002

**Figure 4:**
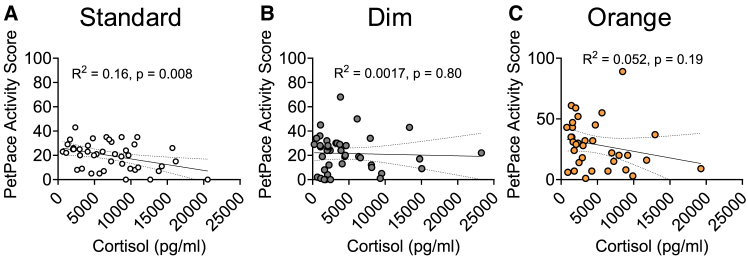
Pearson correlations between cortisol and PetPace activity score (A–C) Pearson correlations examining the relationship between cortisol and PetPace activity scores in (A) standard, (B) dim and (C) orange light. Individual cats are shown as circles, *n* = 45-60 per group. R^2^ and *p* values are indicated next to linear regression lines. Dashed curves indicate 95% confidence intervals.

To understand if increased hiding in the dim light may be limited to when human experimenters were present in the room or if increased hiding was persistent across each day, we analyzed overall activity levels via PetPace collars (Figure 3C; Table 1). PetPace activity scores were only dependent on day in shelter (F[2, 47] = 5.68, *p* = 0.006), with increased activity scores on day 3 (30.0 ± 3.19; *p* = 0.0014) and day 4 (29.7 ± 2.33; *p* = 0.0027) compared to day 2 (21.1 ± 2.41). To understand if changes in activity level were linked with stress response, we conducted correlation analyses of urinary cortisol levels with PetPace activity scores for each light condition (Table 4). PetPace activity and cortisol were only correlated under standard light, where reduced activity was linked to increased cortisol levels, with no significant relationship in dim or orange light (Figure 4).

**Table 4:** Pearson correlation data for urinary cortisol concentration with cat stress scores (CSS) and PetPace activity score for each light condition

Variable	Light Condition	Pearson r	95% confidence interval	R^2^	*p* value
PetPace Activity Score	Standard	**−0.40**	**−0.62 to -0.11**	**0.16**	**0.0084**
	Dim	−0.042	−0.34 to 0.27	0.0017	0.79
	Orange	−0.23	−0.53 to 0.12	0.052	0.20
3 Feet	Standard	**0.45**	**0.18 to 0.70**	**0.21**	**0.0018**
	Dim	**0.39**	**0.12 to 0.60**	**0.15**	**0.0055**
	Orange	0.056	−0.21 to 0.31	0.0031	0.68
Approach	Standard	**0.53**	**0.28 to 0.71**	**0.28**	**0.0002**
	Dim	**0.41**	**0.15 to 0.62**	**0.17**	**0.0028**
	Orange	0.0055	−0.25 to 0.26	<0.0001	0.97
Hand Extended	Standard	**0.62**	**0.40 to 0.77**	**0.39**	**<0.0001**
	Dim	**0.48**	**0.23 to 0.67**	**0.23**	**0.0004**
	Orange	−0.0095	−0.27 to 0.25	<0.0001	0.094

Males and females are combined. Significant data (*p* < 0.05.) are bolded, n = 45–60 per group.

### Shelter cats exhibited circadian rhythms in locomotor activity that did not depend on light condition

**Figure 5:**
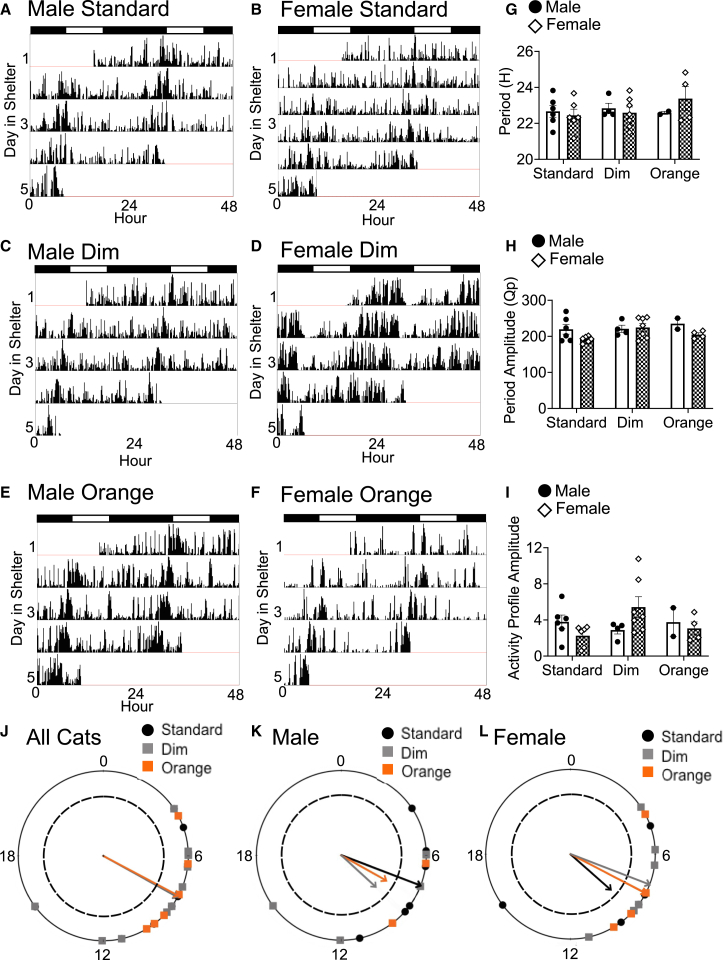
Cat actigraphy under standard, dim, and orange lighting Representative actograms for (A, C, and E) male and (B, D, and F) female cats over days 1–5 in the shelter. (G) Circadian period (H) Chi^2^ amplitude, and (I) activity profile amplitude were analyzed via two-way ANOVAs in standard, dim, and orange lighting, n = 2–7 per group. Data are represented as mean ± SEM. Individual cats are represented by circles (males) or diamonds (females). Acrophase plotted in radians for J all cats, n = 2–7 per group, (K) males, n = 2–6 per group, and (L) females, n = 4–7 per group, in standard (black circle), dim (gray square), and orange (orange square) lighting. Rayleigh tests of uniformity revealed clustering of acrophase, indicated by arrowheads crossing the dashed circle (*p* = 0.05).

As the PetPace-generated activity scores and hiding behavior only represent an averaged or singular point in time, we wanted to gain a higher resolution of locomotor activity patterns across individual cats’ adaptation to the shelter environment. We exported raw data from the PetPace collars to analyze circadian rhythms using ClockLab Analysis 6 (Actimetrics, Wilmette, IL). All cats (*n* = 29) were rhythmic (representative actograms: Figures 5A–5F) and displayed circadian periods ranging from 21.5h to 24.83h (Figure 5G). Period (Male: W = 0.96; *p* = 0.62; Female: W = 0.87; *p* = 0.29), Chi^2^ amplitude (Male: W = 0.81; *p* = 0.15; Female: W = 0.99; *p* = 0.77), and activity profile (Male: W = 0.78; *p* = 0.06; Female: W = 0.93; *p* = 0.48) data passed Shapiro–Wilk test for normality. There were no interactions or main effects of light condition or sex (statistics reported in Table 5) on circadian period, Chi^2^ amplitude (Figure 5H), or activity profile amplitude (Figure 5I). Representative Chi^2^ periodograms depicting peaks of rhythmic activity (Qp values), with the highest peak corresponding to the estimated true period of the time series^65^ and activity profiles are reported for males (Figures S1A–S1F) and females (Figures S1G–S1L) for each light condition.

**Table 5:** Two-way ANOVA for sex differences and light condition for actigraphy data, n = 2–7 per group

Variable	Light Condition	*p* value	Sex	*p* value	Interaction	*p* value
Period	F (2, 23) = 0.45	0.65	F (1, 23) = 0.10	0.75	F (2, 23) = 0.75	0.49
Chi^2^ Amplitude (Qp)	F (2, 23) = 1.65	0.21	F (1, 23) = 3.66	0.07	F (2, 23) = 1.55	0.23
Activity Profile Amplitude	F (2, 23) = 0.88	0.42	F (1, 23) = 0.015	0.90	F (2, 23) = 2.97	0.07

As it is important to understand the activity patterns of domestic cats in a shelter environment as a first step toward understanding how the timing of entraining cues, such as caretaking activities (e.g., feeding, introducing enrichment toys, or social interactions), may impact activity patterns, we determined the chronotype of cats in our study by identifying the acrophase (timing of peak activity), in each light condition. Notably, in the shelter environment for this study, several timing cues (zeitgebers) were present, including shelter staff manually turning on the lights each day at approximately 0800h and off at approximately 1700h, shelter staff administered medications to cats at 0830h (if a second dose was required, it was administered at 1500h), shelter staff cleaned all housing units and fed each cat at 1030h, daily enrichment was given to each cat at 1300–1400h, and researchers conducted daily data collection between 1300 and 1700h. Cat acrophase for males and females combined (Figure 5J) was significantly clustered in standard (2.09rad ±0.59; Rayleigh = 0.72, *p* = 0.001), dim (2.08rad ±0.67; Rayleigh = 0.70, *p* = 0.003), and orange light (2.06rad ±0.44; Rayleigh = 0.87, *p* = 0.005), corresponding to the time of lights on (ZT0, ∼0800h) in the room, and did not differ between light conditions. Watson’s two-sample tests of homogeneity did not reveal differences between acrophase in each light condition (*p* > 0.10). When each sex was examined independently (Figures 5K–5L), acrophase clustering was limited to standard light in males (1.94rad ±0.54; Rayleigh = 0.89, *p* = 0.001), dim light in females (1.94rad ±0.54; Rayleigh = 0.82, *p* = 0.001), and orange light in females (2.05rad ±0.46; Rayleigh = 0.84, *p* = 0.048), with all others groups failing to cluster (*p* > 0.05).

### Cat stress scores were reduced by type of lighting and time in shelter

**Figure 6:**
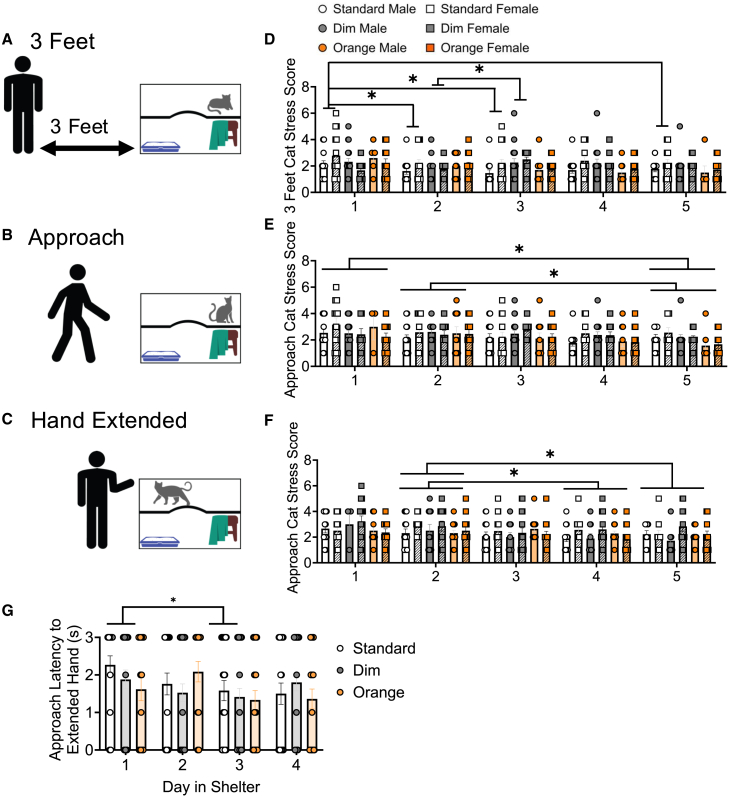
Cat stress score by day in shelter in standard, dim and orange overhead lighting in the 3-task behavioral approach test Cat stress scores (A and D) three feet from the unit, (B and E) approaching the unit, and (C and F) with hand extended, analyzed mixed model ANOVA (Type 3 tests, KR-method), *n* = 10–15 per group. (G) Cat latency to approach in response to an extended hand, measured in seconds, by day in shelter in standard, dim and orange light, analyzed via repeated measures mixed-effects model with Geisser-Greenhouse correction; *n* = 27–41 per group. Data are represented as mean ± SEM. Individual cats are represented by circles. “∗” indicates significant differences (*p* < 0.05).

High stress, as measured by physiological and behavioral outputs, is associated with reduced adoptions in cats^26^ and increased health risks, including respiratory, gastrointestinal, dermatological, and feline interstitial cystitis, among others.^63^ To increase our understanding of cat stress behavior in response to human presence, human approach, and human-initiated interaction, we used a 3-task behavioral approach test (Figures 6A–6C). In brief, an experimenter video recorded the cat as the experimenter 1: stood 3 feet away directly in front of the housing unit for 2 s (Figure 6A; 3-Feet), 2: approached the cat unit at a slow and steady pace (Figure 6B; Approach), and 3: reached a hand toward the cat within the unit, with palm down and fingers extended (Figure 6C; Hand Extended). Cat behavior was scored at all three time-points using a modified version of the validated cat stress score, where 1 is fully relaxed and 7 is terrified (Table S1).^57^ There were no significant three-way interactions for 3 Feet, Approach, or Hand Extended tasks (mixed model ANOVA, Table 6).

**Table 6:** Mixed model ANOVA table (Type 3 tests, KR-method) for sex, day in shelter, and sex differences for each task in the Behavioral Approach Test, *n* = 10–15 per group

Variable	Effect	df	F	*p* value
3 Feet	Sex:Light:Day	8, 266.93	1.33	0.23
	**Light:Day**	**8, 266.93**	**2.94**	**0.004**
	Sex:Day	4, 267.36	0.26	0.91
	Sex:Light	2, 79.76	1.76	0.18
	Day	4, 267.36	2.12	0.079
	Light	2, 79.76	0.29	0.75
	Sex	1, 79.94	0.54	0.46
Approach	Sex:Light:Day	8, 243.00	0.56	0.81
	Light:Day	8, 243.00	1.78	0.082
	Sex:Day	4, 243.40	0.06	0.99
	Sex:Light	2, 72.64	0.68	0.51
	**Day**	**4, 243.40**	**4.07**	**0.003**
	Light	2, 72.64	0.80	0.45
	Sex	1, 72.79	0.36	0.55
Hand Extended	Sex:Light:Day	8, 266.90	0.45	0.89
	Light:Day	8, 266.90	1.49	0.16
	Sex:Day	4, 267.37	0.23	0.92
	Sex:Light	2, 80.23	1.27	0.29
	**Day**	**4, 267.37**	**3.72**	**0.006**
	Light	2, 80.23	0.74	0.48
	Sex	1, 80.48	0.52	0.47

Significant data (*p* < 0.05.) are bolded.

For males and females combined, the 3-Feet task (Figure 6D) revealed a significant interaction of both lighting and day in shelter (F[8, 266.93] = 2.94, *p* = 0.004). In standard light, cat stress scores were reduced on day 2 (1.89 ± 0.22, *p* = 0.0083), day 3 (1.82 ± 0.23, *p* = 0.0019), and day 5 (2.00 ± 0.21, *p* = 0.010), compared to day 1 (2.46 ± 0.29). Cat stress scores were increased on day 3 (2.36 ± 0.18, *p* = 0.025) compared to day 2 (1.90 ± 0.14) in dim light, and there were no differences in 3 Feet cat stress scores in orange light. There were no significant interactions for the approach (Figure 6E) or hand extended (Figure 6F) tasks, and light condition alone did not impact approach or hand extended tasks (Table 6). However, there was a main effect of day in shelter for both the approach (F[4, 243.4] = 4.07, *p* = 0.003) and hand extended (F[4, 267.37] = 3.72, *p* = 0.006) tasks. In the approach task, cat stress scores were higher on day 1 (2.61 ± 0.16, *p* = 0.013) and day 2 (2.46 ± 0.13, *p* = 0.010) compared to day 5 (2.04 ± 0.13).

**Figure 7:**
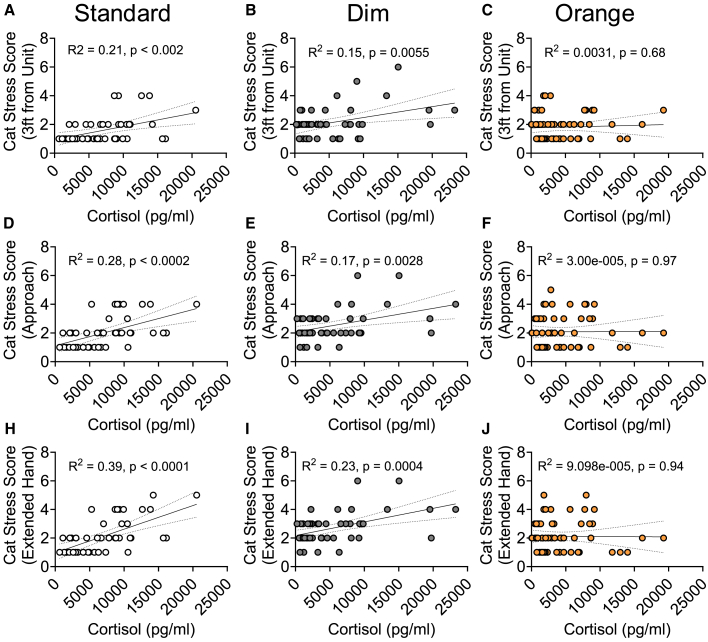
Pearson correlations between cortisol and cat stress scores from the 3-Task Behavioral Approach Test Pearson correlations examining the relationship between cortisol compared to cat stress scores in the (A–C) 3 Feet, (D–F) Approach, and (H–J) Extended Hand tasks. Individual cats are shown as circles, *n* = 45-60 per group. R2 and *p* values are indicated next to linear regression lines. Dashed curves indicate 95% confidence intervals.

In the hand extended task, cat stress scores were higher on day 2 (2.58 ± 0.13) compared to day 4 (2.22 ± 0.11, *p* = 0.049) and day 5 (2.23 ± 0.14, *p* = 0.050). To validate the 3-task behavioral approach test, we compared the cat stress scores to urinary cortisol levels via correlation analyses (Table 4). Higher cat stress scores were positively correlated with increased urinary cortisol levels for all 3 tasks in the behavioral approach test in standard and dim, but not orange light (Figure 7).

As social affiliation toward humans is a strong indicator of successful adoption, we also measured latency to approach an extended human hand. Light condition did not impact latency to approach (*p* = 0.73). As cats spent increased time in the shelter, the latency to approach decreased (*p* = 0.05), such that cats had reduced approach latencies on day 3 (mean = 1.52, *n* = 77) compared to day 1 (mean = 1.94, *n* = 77) in the shelter (mean difference = 0.42, standard error of difference = 0.18, 95% CL of difference = −0.0434 to 0.875, adjusted *p* = 0.09).

## Discussion

This study investigated the ability of light intensity and composition as well as length of time in the shelter to modulate cat stress and locomotor circadian rhythms. A major focus of the field of circadian biology has been advancing human health; however, increasing our understanding of circadian mechanisms guiding health across species has important implications beyond humans, including the potential to improve non-human animal health and welfare, recently reviewed by Farag et al., 2024.^66^ Here, we found that both male and female shelter cats had short (22-23h) circadian rhythms in locomotor activity that peaked at the time of lights on, independent of light condition. Additionally, light intensity, light composition, and the length of time spent in the shelter, interreacted to modulate cortisol, stress behaviors, and overall activity patterns. Surprisingly, we did not observe any sex differences, although it is possible that sex-specific effects might emerge with increased sample sizes. Together, the data presented here characterize circadian locomotor activity patterns in shelter cats and demonstrate that both lighting conditions and number of days in the shelter can reduce physiological and behavioral measures of cat stress.

Understanding the circadian rhythms in physiology and behavior of cats in a shelter environment is an important first step toward determining how the timing of care, such as food, social interactions with staff and conspecifics, and medications, should be coordinated to ensure peak health of the cats. Studies examining circadian rhythms in domestic cats have been conducted since the early 1960s, where male cats were found to have a circadian peak in mean heart rate at the time of lights on.^39^ However, there are varying reports on the activity patterns in domestic cats, ranging from evidence suggesting a lack of circadian activity patterns to reports of nocturnal, crepuscular, and diurnal circadian activity rhythms.^27^^,^^28^^,^^29^^,^^30^^,^^31^ Previous work found that cats in constant darkness exhibit free-running circadian rhythms,^67^ but there are mixed reports that include circadian,^1^^,^^31^^,^^68^ polyphasic,^32^^,^^33^ and a lack of circadian rhythms^32^^,^^69^ in standard light dark cycles. Here, we find that shelter cats displayed circadian rhythms in behavioral activity with ∼22-23h periods in all light conditions. Our data support the current hypothesis that cats exhibit a high variability in activity patterns in controlled housing conditions^1^ and that the environment exerts a strong influence on the expression of cat locomotor activity patterns.^31^^,^^32^^,^^34^^,^^35^^,^^36^^,^^37^^,^^38^ Notably, in our study, cats were housed under an extended dark period (9h light: 15h dark). Photoperiod has not been found to modulate cortisol levels in cats,^70^ making it unlikely that the extended dark phase alone impacted stress hormone levels. However, others have shown that cats can rapidly adapt their activity patterns to light cycles in a laboratory environment,^68^ and it is possible that the shorter activity periods found here are driven by re-entrainment as the cats adapt to a new lighting schedule in their shelter environment. This work was limited by the shelter’s designated quarantine/holding period for all intact cats, as spay/neuter procedures occurred after 5 days in the shelter, but it will be of interest for future studies to lengthen the time of observation to understand if the less-than-24h periods are innate or a function of re-entrainment.

Two of the main factors guiding cat activity patterns are proximity to humans and feeding, with several reports documenting circadian activity pattern entrainment to both human activity and feeding schedules.^32^^,^^37^ Interestingly, our data find that shelter cat acrophase occurs at the time of lights on. This suggests that cats may be entrained to the timing of lights on compared to the timing of food availability, which occurred approximately 2.5 h after cat peak activity. However, one limitation of our study is the inability to tease apart the potential interactions of shelter cat activity patterns in relation to zeitgeber availability. Notably, the shelter lights were not coordinated by an automatic timing system, instead the lights were manually turned on by shelter staff as they entered the room for morning health checks. It is possible that acrophase is impacted not only by the timing of light, but is also influenced by human staff presence, although additional work will be needed to discern how these variables impact the cat’s temporal niche. Importantly, this work suggests that daytime care (feeding, interactions with staff, medication administration) is not likely to introduce health risks associated with desynchrony, as cats in this cohort adapted activity patterns to correspond with these activities. However, more controlled studies are needed to determine if specific timing of care and enrichment, such as a consistent feeding and treat schedule, is more beneficial compared to scattered care, which would provide a more naturalistic environment. This is a potentially important advancement for the timing of care for other environments that house domestic cats, such as boarding, veterinary, and research facilities.

In addition to locomotor activity patterns, light quality has been demonstrated to impact mood and stress in mammals.^2^^,^^7^^,^^8^^,^^9^^,^^10^^,^^11^^,^^12^^,^^13^ In domestic cats, both transition-related increases in stress, including entering a new housing enviornment,^57^ and the animal shelter environment^25^ are well-established stressors, making the initial days following intake into an animal shelter an especially vulnerable time. Here, we examined cats housed in the shelter’s windowless quarantine and holding room that was used to house recently admitted cats, separate from the shelter’s cat adoption room and not open to the public. This allowed for a highly controlled environment to understand how light qualities impact a cat’s transition into a shelter. Our study confirms prior work that time in a new housing location is a critical factor for stress reduction in cats,^57^^,^^71^ as we found that both physiological stress (urinary cortisol) and behavioral indicators of stress (cat stress scores, 3-task behavioral approach test) decreased across days in the shelter in all light conditions. A longer length of time in shelter (i.e., the more days) could increase the likelihood of a cat to become habituated to their surroundings, including their housing unit, other cats in the room, and shelter staff. The reduced cat stress scores across the 3-task behavioral approach test reported here are consistent with previous work that found significant reductions in behavioral cat stress scores after 5 days in a cattery^57^ or animal shelter.^71^ We found that like cortisol and locomotor activity, cat latency to approach an extended human hand depended on the length of time in the shelter. Specifically, cats under all light conditions had decreased latency to approach on day 3 in the shelter.

Interestingly, while urinary cortisol decreased across days in both standard and dim lighting conditions, the dim, orange (blue-depleted) light condition decreased urinary cortisol concentration in both male and female cats to a greater extent than standard light on later days (days 5). This could indicate faster adjustment to the shelter housing conditions, but more work, such as including an extended duration of data collection greater than 5 days, will be necessary to better understand if these effects are due to habituation or potential downregulation of cortisol as a result of chronic stress.^72^ Interestingly, stressed cats are known to suppress active exploratory and play behaviors,^73^ making it possible that increases in activity levels were linked to reduced stress; however, increased activity and reduced urinary cortisol were only significantly correlated under standard light (Figure 7). This demonstrates an important avenue for future work to examine the mechanisms driving changes in locomotor and stress behaviors and the nuances in light qualities that may modulate such stress-related behaviors.

In addition to reduced activity, cats naturally hide as a stress response and will even overturn litter boxes to create hiding spaces if there are no other alternatives.^63^^,^^64^ In the present study, all cats were provided with a hiding space within their unit. We found that light impacted cat hiding behavior, where cats had an increased probability of hiding under the dim light, but not during standard or dim, orange lighting. This provides additional evidence that light can modulate stress-related behaviors in cats, where dim light is less effective at reducing cat stress, compared to standard light and dim, orange light. While hiding boxes have been a significant stress-reduction intervention for shelter cats, hiding boxes do not prevent physiological results of stress such as weight loss^74^ or improve a cat’s chances of adoption,^75^ suggesting that cats may need multiple stress-reduction interventions to be fully comfortable in shelter environments. While additional investigation is needed to tease apart the complex relationships between the stress variables examined here, taken together our data suggest that urinary cortisol, locomotor activity, hiding, and approach behaviors depend on both light manipulation and time spent in the shelter. As such, care should be taken in the design and documentation of lighting conditions in domestic cat housing facilities and research studies. Additionally, given the high genetic and behavioral overlap of domestic cats and their wild relatives,^19^^,^^20^ this work is likely to be relevant for conservation research. Light quality may be especially important to consider for wild Felidae species that are undergoing captive breeding and assisted reproductive efforts, where precise circadian timekeeping is essential for successful reproduction, and high stress can impair breeding efforts.^21^

Together this work demonstrates for the first time that room light manipulation, in conjunction with the length of a shelter stay, may help shelters enact easy and inexpensive changes that make the adjustment period shorter and less stressful for incoming cats. Here, we also define the activity patterns of male and female domestic cats in a shelter environment as a first step toward developing recommendations for the timing of caretaking activities. These results highlight the importance of considering light quality as an important factor for stress management when designing or modifying cat housing.

### Limitations of the study

In addition to the limitations of this study characterized throughout the Discussion, cat ownership history prior to study enrollment was not available, making it impossible to distinguish between cats that were surrendered by owner or entered the shelter as strays, which might impact stress levels upon arrival at the shelter. It is possible that variability in our data could be explained by combinations of feral and non-feral cats, or that some of our findings, such as increased activity profile amplitude in female under dim light, might reflect an instinctual hunting response from feral populations. To generate sufficient data for analysis, we enrolled cats ranging from 8 months to 6 years, which encompasses two age ranges, junior and prime,^76^ and thus it is possible that our data may be complicated by potential age effects. A limitation of the 3-task behavioral approach test is that the cat stress scoring system does not discriminate between stress (negative high arousal) and “excitement” (positive high arousal). For example, a cat with high affiliation for humans, a desirable trait for adoption, that stood and approached an experimenter could not be categorized as “fully relaxed”. To account for this, we included the latency to approach dataset, which measures a cat’s motivation to interact with a human and is an established marker of feline affiliation toward humans.^75^^,^^77^ In an effort to minimize interactions between researchers and enrolled cats, we did not collect light data from underneath the covered stool in each cat unit; however, it is possible that variations in blanket/towel thickness, resulting in a range of dim-dark hiding spaces, coupled with the amount of time cats spent hiding (not tracked in this study), could impact the results. Sample sizes vary between different analyses due to missing data and technical challenges. Several cats did not use the litter pan, and animals that did not give three samples of urine during the testing period were excluded from cortisol analyses. Cats were sometimes not present in their unit at the time of behavioral data collection due to removal from their unit by shelter staff, and no behavioral task data for those days were collected. Missing data from the PetPace collars, included data lost via Wi-Fi disconnection, cat collar self-removal, and inability of experimenters to safely place a collar on a cat. It is also important to note that for unknown reasons the shelter had reduced intake of male cats during the data collection periods for orange lighting (July–August 2021 and April–October 2022), which resulted in lower sample size for some datasets. Efforts were made to distribute data collection across seasons; however, due to the reduced intake of male cats during the second timepoint for orange lighting, there is a significant overlap of summer months. It is possible that our results may be linked to time-of-year or seasonal effects, although more work is needed to confirm this.

## Resource availability

### Lead contact

Requests for further information and resources should be directed to and will be fulfilled by the Lead Contact, Alexandra Yaw (yawalexa@msu.edu).

### Materials availability

This study did not generate new unique reagents.

### Data and code availability

• All data reported in this paper will be shared by the lead contact upon request.
• This paper does not report original code.
• Any additional information required to reanalyze the data reported in this paper is available from the lead contact upon request.

## Acknowledgments

Thank you to our partners at the Ingham County Animal Control and Shelter and undergraduate researchers Grace Jaksen, Jessica Crane, Kathryn Hurt, Skyler Mack, and Eleanor Vondette for their assistance in data collection. Research reported in this publication was supported in part by grants from 10.13039/100011138MSU AgBioResearch and 10.13039/100007709Michigan State University to J.J. and H.M.H. A.M.Y. was supported by the 10.13039/100009633Eunice Kennedy Shriver National Institute of Child Health & Human Development of the National Institutes of Health under Award Numbers F32HD107852 and K99HD113843, and the Resident/Fellow Award from the MSU’s College of Veterinary Medicine (CVM) Feline Health Endowment, with additional support of training to A.M.Y. by T32HD087166. The content is solely the responsibility of the authors and does not necessarily represent the official views of the supporting institutions.

## Author contributions

Conceptualization: A.M.Y., J.J., and H.M.H.; methodology: A.M.Y., M.E.G., J.J., and H.M.H.; investigation: A.M.Y. and M.E.G.; writing – original draft: A.M.Y. and M.E.G.; writing – reviewing and editing: A.M.Y., M.E.G., J.J., and H.M.H.; funding acquisition: J.J. and H.M.H.; resources: J.J. and H.M.H.; supervision: A.M.Y., J.J., and H.M.H.

## Declaration of interests

The authors declare no competing interests.

## STAR★Methods

### Key resources table

**Table:** 

REAGENT or RESOURCE	SOURCE	IDENTIFIER
**Experimental models**		

Domestic Cats (*Felis catus*)	Ingham County Animal Control and Shelter	N/A

**Software and algorithms**		

CIELUV chromaticity diagram 1976	E H H Hasabeldaim 2021	https://sciapps.sci-sim.com/CIE1976.html
ClockLab	Actimetrics, Lafayette Instrument Company	https://actimetrics.com/products/clocklab/
PetPace	PetPace LLC	https://petpace.com
Prism	GraphPad, Dotmatics	https://www.graphpad.com

### Experimental model and subject details

All animal procedures were performed according to protocols approved by the Institutional Animal Care and Use Committee of Michigan State University. We tested 101 domestic cats (*Felis catus*; 48 male and 53 female), residing at a Midwestern animal control and shelter from March 2021 through October 2022 (Figure 1C). Intact healthy cats between the ages of 8 months – 6 years, corresponding to junior (7 months-2 years; 59 cats, 21 male) and prime (3-6 years; 42 cats, 27 male) aged cats, according to the American Animal Hospital Associate and American Association of Feline Practitioners (AAHA/AAFP) Feline Life Stage Guidelines,^76^ that were housed individually and under ownership by the shelter were enrolled in the study. No cats underwent surgical procedures while they were enrolled in the study, including spay/neuter procedures. Cat exclusion criteria included: documented injuries, including eye damage or blindness, pregnancy or recent birth (∼10 weeks), or co-housing. Cat ownership history prior to study enrollment was not available. All sample and animal behavior data were collected at Ingham County Animal Control and Shelter located in Mason, Michigan. Subjects were housed in the shelter’s quarantine and holding room that was used to house recently admitted cats, separate from the shelter’s cat adoption room and not open to the public. The housing room (Figure 1A) was 17.6 m^2^ and windowless, with accessibility by one door. The housing room was located on the interior keycard access portion of the building, away from dog adoption kennels and associated outdoor spaces. The housing room shared walls with the adoption lobby, office spaces, and a dog quarantine and holding room. Ambient noise levels were 62.7-74.5dB measured by a TOPTES (TS-501A) Sound Level Meter (Guangzhou Zhongguoqi Technology Co., Ltd., Guangdong, China). The room housed 22 units (10 on one wall, 12 on opposite wall) for cat containment. There were two sizes of housing units, large units were 0.68 m^3^ and small units 0.55 m^3^ (Figure 1A). Cats were placed in individual housing units at random by the shelter staff. Each unit contained an upper and lower level (Figure 1B). The upper level was left empty as open housing space. The lower level contained: food and water bowls, bedding (blankets or towels), a litter pan, and a stool covered by fabric allowing cats more privacy and a hiding space. Shelter staff provided a variety of daily enrichment activities for the cats, including toys, handling, and/or treats and enrichment type was varied as resources permitted. The holding room was only accessed by shelter staff (for feeding, enrichment, cleaning, medical checks, moving cats) and our researchers (for data collection). Lights were turned on and off manually daily by shelter staff, with lights on at approximately 0800h and off at approximately 1700h. Each day, staff administered medications to cats at 0830h (if a second dose was required, it was administered at 1500h). At 1000h the staff then cleaned all kennels and fed every cat. Finally, cats received daily enrichment around 1300-1400h. Researchers conducted all enrollment and data collection between 1300-1700h. Interactions between researchers and cats were minimal. Researchers were instructed to act as neutral observers, and only interacted (physical or verbal) with cats during PetPace collars application and removal in addition to litter pan exchanges. Animal health was monitored regularly by on-site veterinary staff.

Light conditions in the shelter room were varied across the 19 months of testing (Figure 1C) and cats were assigned to a light condition if they maintained the same light for all days of testing. Enrolled cats were monitored with data collection for five days (Figure 1D). Cat’s enrollment occurred within the first 24h in the shelter. On the day of enrollment, initial approach test videos were filmed before cats were given a new litter pan containing hydrophobic litter and fitted with a PetPace collar (PetPace, Burlington, MA). For the next four days, activity data were continuously collected via PetPace smart collars, the approach test was conducted and filmed each day prior to urine samples collection. Cats were unenrolled on the fifth full day in the study.

#### Apparatus/materials

There were three light conditions: standard (control, unaltered shelter LED lighting), dim, and orange (dim, blue-depleted) light (Figure 2). Light filters (B & H Foto & Electronics Corp, New York, NY) were placed over the LED overhead lights (standard light) to achieve dim (BH #ROE250S: MFR #102302502124) and orange lighting (BH #ROE204S: MFR #102302042124). Light qualities in cat units (both upper and lower levels) were measured with UV Illuminance Spectrophotometer SRI-2000 UV (Allied Scientific Pro™, Québec, Canada) at1300h. The CIELUV chromaticity diagram 1976 (Figure 2A) was created through online open-source software.^78^ As multiple aspects of light quality are known to modulate physiology and behavior,^2^^,^^7^^,^^8^^,^^9^^,^^10^^,^^11^^,^^12^^,^^13^ we analyzed both light intensity and composition (Figure 2). As light composition can impact mood, a CIELUV 1976 chromaticity diagram for the average human-perceived lighting color in each of the light conditions was generated (Figure 2A). Both human perceived wavelength (λp; main effect of lighting F(2, 5) = 59.22, *n* = 8, *p* = 0.0003) and dominant wavelength (λd; main effect of lighting F(2, 5) = 465.6, *n* = 8, *p* < 0.0001) were increased in dim, orange lighting compared to both standard and dim (Figures 2C–2E). Light intensity (Figures 2B and 2E) was reduced in both dim and orange lighting compared to standard (main effect of lighting F(2,10) = 7.783, *n* = 8, *p* = 0.009) and the lower level of the cat unit had reduced light intensity compared to the upper level (main effect of unit level F(2,10) = 18.34, *n* = 8, *p* = 0.002). To monitor room light timing, a three-day continuous recording with sampling every 5 min were collected in each lighting condition via HOBO Pendant MX2202 Temperature/Light Data Recorder (Figure 2B). Light condition differences are reflected in the representative spectral power distributions (Figures 2F–2H), where orange lighting has a reduced peak in the blue-light portion (450-495nm) that is not present in standard or dim spectral power distributions and standard light intensity (1.24 mW/m^2^) is higher than dim (0.93 mW/m^2^) and orange light (0.82 mW/m^2^).

### Method details

#### Urinary cortisol

Cortisol was used as a physiological indicator of stress. Urine was the chosen medium for obtaining this hormone, as drawing blood may have imparted more stress on the cats. Cats received a fresh litter pan containing hydrophobic cat litter, Nosorb (Catco Vet Products Inc., Florida, USA) or Kit 4Kat (Coastline Global Inc., Pennsylvania, USA), daily. Urine was collected once daily from litter pans, as previous work demonstrated that litter urine samples collected 24h following a stressor reflect increases in cortisol levels similar to needle drawn serum and urine.^79^ Urine samples were immediately frozen at −20°C. Cortisol was analyzed via Cortisol ELISA kit, ADI-900-071; RRID: AB_3697107, (ENZO Life Sciences INC., Farmingdale, NY). Urine samples were thawed at room temperature for 24h before steroid displacement and added to the plate at a 1:10 dilution and run in duplicate. Animals that did not give three samples of urine for the 4-day testing period were excluded from analysis. Data include n = 4–12 per group.

#### Locomotor actigraphy

PetPace SmartCollars (PetPace LLC, Massachusetts, USA) were used to record activity data every two minutes for the length of a cat’s enrollment. All data were stored wirelessly in the online PetPace Health Monitoring Portal. PetPace Activity scores were calculated through a proprietary scale within the PetPace software and represented the combined calculated values of activity intensities, activity frequency and duration.^80^ Individual activity counts were exported from the Health Monitoring Portal and converted to be compatible with ClockLab Analysis 6 (Actimetrics, Wilmette, IL). Data were analyzed in 10 min bins and daily onset and offset of activity, defined as a period of 4h of activity following 4h of inactivity (onset) or a period of 4h of inactivity following 4h of activity (offset). Chi^2^ periodograms^81^ were generated from 19 to 28h, reflecting the general biological designation for circadian rhythms^82^ with significance set at 0.005. All cats that exhibited a significant peak were deemed rhythmic, with the maximum amplitude reported as Qp. Activity profiles were generated using the circadian period (tau) estimated from the Chi^2^ periodogram for the same testing dates. Acrophase was generated using at least three days of activity. Missing data included data lost via Wi-Fi disconnection, cat collar self-removal, and inability of experimenters to safely place a collar on a cat. Data include n = 2-7 per group.

#### 3-Task Behavioral Approach Test and approach latency

The daily 3-Task Behavioral Approach Test (Figure 5, Figure 6) was conducted prior to all other daily sample collections (Figure 1D) and was recorded via GoPro HERO7 Black camera (GoPro Inc., San Mateo, California). Each task of the approach test (3 feet, approach, hand extended) took place for 3 sec., with a total of ∼9sec. to complete the test, similar to the approach test in Arhant & Troxler, 2017.^83^ To begin, the experimenter stood 3 feet away (∼91cm) and directly in front of the housing unit and began video recording the cat while remaining still. After 3 sec, the experimenter approached the cat unit at a slow and steady pace (∼1 ft/s; ∼0.3 m/s) to complete the approach task. Next, the experimenter reached a hand towards the cat within the unit, with palm down and fingers extended, and the hand remained extended for 3sec. before finishing the recording.

Videos were scored for stress using an adapted cat stress score (CSS) system^56^^,^^57^ by experimenters who were blind to the groups. The CSS (Table S1) identifies behavioral signs of stress on a scale of one to seven, with a score of one equating to a fully relaxed cat (laid out on back or side, eyes closed or half open, resting or sleeping) while a score of seven describes a terrified cat (fast breathing, shaking, dilated pupils, ears flattened back). Cats located beneath the stool and towel during any task of the behavioral approach test were deemed “not visible” for that portion of the task. Cats that remained under the stool and towel during all three tasks were deemed “hiding,” not included in CSS data, and were used to calculate the percentage of cats hiding. Approach latency was defined as the number of seconds a cat took to approach the front of the cat unit during the hand extended portion of the test. Data from cats removed from their unit by shelter staff during the time of behavioral data collection were not included. Data include n = 4-19 per group.

### Quantification and statistical analysis

Data were analyzed using GraphPad Prism 8 (Version 10.4.1 (532), Graph Pad Software, La Jolla, CA) and RStudio (Version 2024.12.1 + 563 (2024.12.1 + 563)). Significant differences were designated as *p* < 0.05 and data are reported as mean ± standard error mean (SEM). In unit light quality data (peak wavelength, dominant wavelength, and light intensity), approach latency, and hiding data were analyzed by two-way Analysis of Variance (ANOVA). Room light intensity was analyzed via a two-way repeated-measures ANOVA, followed by Tukey’s multiple comparison test. Cortisol, PetPace activity scores, and cat stress scores (3-Trial Behavioral Approach Test) were analyzed by linear mixed-effects models with sex, light condition, and day as within subject factors. Cat IDs were included as a random effect to account for repeated measures across conditions and *p*-values were computed with Kenward-Roger approximation for degrees of freedom to account for missing data. Hiding behavior was analyzed via Bayesian logistic regression model using the brms package in R, which interfaces with Stan for Bayesian inference via Hamiltonian Monte Carlo sampling. The model included all main effects and interactions between sex, light condition, and day in shelter, as well as random intercepts for each individual cat to account for repeated measures. Model convergence was assessed via the R-hat statistic (all values = 1.00), and the effective sample size for each parameter was sufficient. Posterior predictive checks were performed to ensure model fit. Predicted values and 95% credible intervals for hiding were calculated for each combination of sex, light condition, and day in shelter using the model’s posterior distributions. Chi^2^ amplitude and activity profile amplitude were analyzed by two-way ANOVA. Correlation analyses were completed using Pearson r. Post hoc tests were completed using Tukey’s multiple comparison test. All data passed normality testing. Acrophase data were analyzed in R Studio via Rayleigh test of uniformity and 1-criterion analysis of variance for circular data, followed by pairwise comparisons, and Watson’s 2-sample test of homogeneity using Bonferroni’s correction to accommodate familywise error rate, where appropriate. Phase data were reported in radians with circular mean deviation. Statistical analyses for outliers (Grubbs’ test) were conducted on all datasets, and no outliers were identified. All data except for period passed testing for equal standard deviations.
