# Supplementary material for: Light quality and time in shelter modulate behavior and cortisol in the domestic cat (Felis catus)
Source: iScience. 2025 May 20;28(6):112709. doi: 10.1016/j.isci.2025.112709 (PMC12182317; doi:10.1016/j.isci.2025.112709)
Supplement: Document S1. Figures S1 and Table S1 [file mmc1.pdf]

iScience, Volume *28*

Supplemental information

Light quality and time in shelter modulate behavior and cortisol in the domestic cat (*Felis catus*)

Alexandra M. Yaw, Mary E. Gardella, Jacquelyn Jacobs, and Hanne M. Hoffmann

Supplemental Figures and Tables

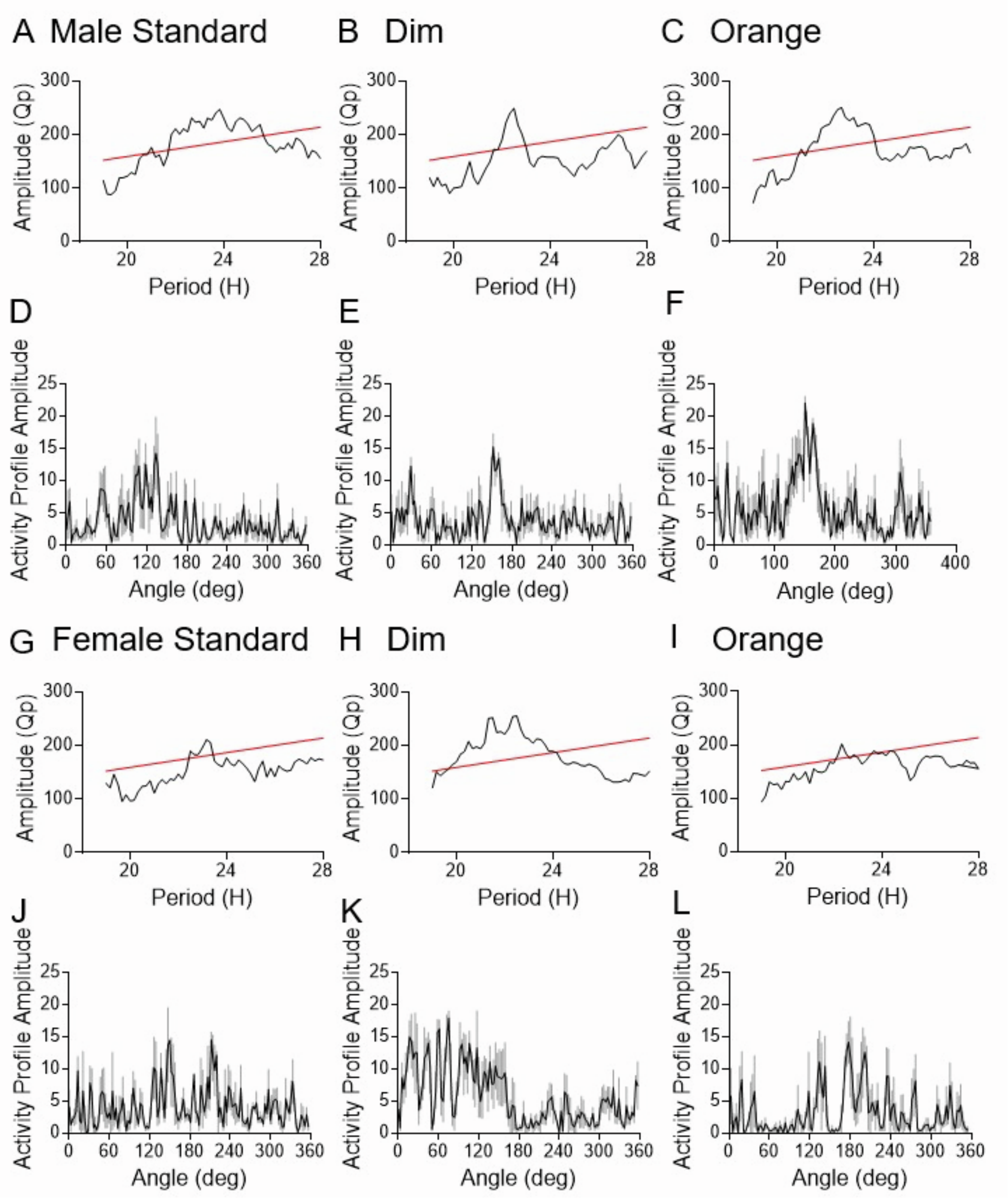

Supplemental Figure 1. Male and female cat actigraphy under standard, dim, and orange lighting. **A-C, G-I)** Representative Chi^2 periodograms and **D-F, J-L)** activity profiles for days 2-5 in the shelter.

Supplemental Table 1. Modified cat stress score rubric adapted from Kessler and Turner, 1997. "I" indicates criteria for an inactive cat and "A" indicates criteria for an actively moving cat.

Score	1. Fully Relaxed	2. Weakly Relaxed	3. Weakly Tense	4. Very Tense	5. Fearful, Stiff	6. Very Fearful	7. Terrified
**Body**	Laid out on side or on back	I: Laid ventrally or half on side or sitting<br>A: standing or moving, back horizontal	I: laid ventrally or sitting<br>A: standing or moving, body behind lower than in front	I: Laid ventral, rolled or sitting<br>A: standing or moving, body behind lower than in front	: laid ventrally or sitting<br>A: standing or moving, body behind lower than in front	I: laid ventrally or crouched directly on top of all paws, may be shaking<br>A: whole body near to ground, crawling, may be shaking	Crouched directly on top of all fours, shaking
**Stomach**	Exposed, slow ventilation	Exposed or not, slow or normal ventilation	Not exposed, normal ventilation	Not exposed, normal ventilation	Not exposed, normal or fast ventilation	Not exposed, fast ventilation	Not exposed, fast ventilation
**Legs**	Fully extended	I: bent, hind legs may be laid out<br>A: when standing, extended	I: bent<br>A: when standing, extended	I:bent<br>A: when standing, hind legs bent in front extended	I: bent<br>A: bent near to surface	I: bent<br>A: bent near to surface	Bent
**Tail**	Extended or loosely wrapped	I: extended or loosely wrapped<br>A: up or loosely downwards	May be twitching<br>I: on the body or curved backwards<br>A: up or tense downwards	I: close to the body<br>A: tense downwards or curled forward, may be twitching	I: close to the body<br>A: curled forward close to body	I: close to the body<br>A: curled forward close to body	Close to the body
**Head**	Laid on surface with chin up or on surface	Laid on surface or over the body, some movement	Over the body, some movement	Over the body or pressed to body, little or no movement	On the plane of body, less or no movement	Near to surface, motionless	Lower than body, motionless
**Eyes**	Closed or half open, may be	Closed, half opened or	Normal	Widely open or pressed together	Widely opened	Fully opened	Fully opened

	blinking slowly	normal opened					
**Pupils**	Normal	Normal	Normal	Normal or partially dilated	Dilated	Fully dilated	Fully dilated
**Ears**	Half-back (normal)	Half back or erected to front or back and forward on head	Half back or erected to front or back and forward on head	Erected to front or back, or back and forward on head	Partially flattened	Fully flattened	Fully flattened back on head
**Whiskers**	Lateral (normal)	Lateral or forward	Lateral or forward	Lateral or forward	Lateral or forward or back	Back	Back
**Vocal**	None	None	Meow or quiet	Meow, plaintive meow or quiet	Plaintive meow, yowling, growling or quiet	Plaintive meow, yowling, growling or quiet	Plaintive meow, yowling, growling or quiet
**Activity**	Sleeping or resting	Sleeping, resting, alert or active, may be playing	Resting awake or actively exploring	Cramped sleeping, resting or alert may be actively exploring, trying to escape	Alert, may be actively trying to escape	Motionless, alert or actively prowling	Motionless
